# Association between CHA_2_DS_2_-VASc score and left atrial function by speckle-tracking echocardiography in patients with sinus rhythm

**DOI:** 10.1186/s44348-026-00090-9

**Published:** 2026-09-16

**Authors:** Kareem Mahmoud, Khaled Mostafa, Sameh Bakhoum, Heba ElDeeb

**Affiliations:** Department of Cardiology, Faculty of Medicine, Cairo University, Cairo, Egypt

**Keywords:** Heart Rhythm, Echocardiography, Heart atria, Risk Assessment

## Abstract

**Background:**

The CHA_2_DS_2_-VASc score is an established tool for thromboembolic risk assessment in atrial fibrillation. Individual components of the score may affect left atrial (LA) function. Speckle-tracking echocardiography (STE) enables detection of early, subtle changes in the LA. This study aimed to test whether LA function, as assessed by STE, differs among patients with sinus rhythm according to their CHA_2_DS_2_-VASc score.

**Methods:**

This study included 150 patients with sinus rhythm. They were grouped by CHA_2_DS_2_-VASc score: low (1 for women, 0 for men), moderate (2 for women, 1 for men), and high (≥ 3 for women, ≥ 2 for men), with 50 patients per group. All participants gave written consent, provided medical histories, underwent a 12-lead electrocardiogram, and received standard echocardiography and STE.

**Results:**

The high-risk group had significantly lower LA reservoir strain (LASr) and LA conduit strain (LAScd) compared to the moderate- and low-risk groups (high vs. moderate vs. low: LASr, 33.2 ± 7.1 vs. 36.9 ± 6.1 vs. 40.6 ± 5.6; LAScd, 20.3 ± 5.7 vs. 23.5 ± 4.8 vs. 26.9 ± 3.9), with all P-values of < 0.01. Moderate-risk patients also showed lower strain values than low-risk patients. In contrast, LA maximal volume index did not differ among the groups (*P* = 0.536). Mediation analysis showed that the association between the score and LAScd was statistically mediated by left ventricular (LV) diastolic dysfunction (89.7%) with no significant direct association (*P* = 0.342). For LASr, 62.4% of the association was statistically mediated by LV diastolic dysfunction, with a residual direct statistical association remaining (β = –0.139, *P* = 0.008).

**Conclusions:**

Increased CHA_2_DS_2_-VASc scores correlated with reduced LASr and LAScd. The association with LAScd was mainly mediated by LV diastolic dysfunction. A direct statistical association of the score was noted with LASr, indicating possible early LA pathology. Further research should assess the clinical relevance of these echocardiographic findings for risk stratification in patients with high CHA_2_DS_2_-VASc score.

## Background

Atrial fibrillation (AF) substantially increases the risk of cardioembolic stroke, contributing to considerable illness and death among affected individuals [1]. Multiple clinical scoring systems have been created to help predict the likelihood of stroke in patients with nonvalvular AF [2, 3]. The CHA_2_DS_2_-VASc score is the most widely used [4, 5]. This scoring system assigns points for a variety of clinical factors [6]:

1. Congestive heart failure, including clinical symptoms or evidence of significant left ventricular (LV) dysfunction, or hypertrophic cardiomyopathy (1 point)
2. Hypertension or the use of antihypertensive medication (1 point)
3. Age: 1 point for ages 65 to 74 years, and 2 points for those 75 years or older
4. Diabetes mellitus, defined as use of oral hypoglycemic agents, insulin, fasting blood glucose ≥ 126 mg/dL (7 mmol/L), or hemoglobin A1C ≥ 6.5% (1 point)
5. Prior stroke, transient ischemic attack, or systemic thromboembolism (2 points)
6. Vascular disease, such as significant coronary artery disease (CAD) by angiography, previous myocardial infarction, aortic plaque, or similar conditions (1 point)
7. Female sex, which acts as a stroke risk modifier rather than a direct risk factor (1 point)

Although designed for AF patients, this score also predicts thromboembolic events in those without AF, such as heart failure, chronic obstructive pulmonary disease, and post–coronary artery bypass grafting (CABG) [7–10]. It can also predict AF development in diabetes, systemic lupus erythematosus, myocardial infarction, and after cardiac surgery [11–16]. The CHA_2_DS_2_-VASc score is associated with other adverse events, such as failed reperfusion after thrombolytic therapy, the no-reflow phenomenon, and contrast-induced nephropathy [17–19]. Although the CHA_2_DS_2_-VASc score is commonly applied, its direct association with subtle abnormalities in left atrial (LA) function among individuals in sinus rhythm remains uncertain. The individual components of the CHA_2_DS_2_-VASc score are known to precipitate LV diastolic dysfunction. In turn, LV diastolic dysfunction can impair LA function, particularly during the conduit phase. However, it remains unclear whether the CHA_2_DS_2_-VASc score has a direct effect on LA function independent of LV diastolic impairment, or whether its association is entirely mediated by LV diastolic dysfunction. This highlights a need for further research.

The LA is essential for effective LV filling, serving as a reservoir, a passive conduit, and an active contractile chamber [20, 21]. Speckle-tracking echocardiography (STE) allows early detection of LA functional changes, even before LA enlargement [22].

There is limited research examining the relationship between LA function and CHA_2_DS_2_-VASc score in individuals with sinus rhythm. The present study evaluated whether this risk score is associated with lower LA strain function prior to LA enlargement, using STE, to inform assessment and early intervention in patients without overt AF.

## Methods

### Ethics statement

The study was approved by the Institutional Review Board of Cairo University (No. MS-108–2022). Written informed consent was obtained from all patients before the procedure for their participation in the study. All study procedures were conducted in accordance with the principles of the Declaration of Helsinki [23] and reported according to the STROBE (Strengthening the Reporting of Observational Studies in Epidemiology) checklist [24].

### Study design

This cross-sectional observational study enrolled both male and female participants with different CHA_2_DS_2_-VASc scores; all were in sinus rhythm. The study ran from May 1 to October 30, 2022. We recruited 150 patients and grouped them by CHA_2_DS_2_-VASc score: low (1 for women, 0 for men), moderate (2 for women, 1 for men), and high (≥ 3 for women, ≥ 2 for men). At the time of study design and data collection (May–October 2022), the CHA_2_DS_2_-VASc score was the recommended risk stratification tool according to current guidelines. Exclusion criteria included AF or atrial flutter, infective endocarditis, significant valvular heart disease, presence of prosthetic valves, pacemakers, poor echocardiographic windows, or LV ejection fraction (LVEF) below 50%.

### Study setting

All patients underwent the following assessment. A comprehensive medical history was obtained for each participant, with particular attention to CHA_2_DS_2_-VASc risk elements including heart failure, age, diabetes, previous stroke, vascular disease, and sex [6]. A standard 12-lead surface electrocardiogram (ECG) was used to assess cardiac rhythm and heart rate. Two experienced cardiologists performed conventional transthoracic echocardiography using a Philips Affinity 50 with an S5-1 transducer (Philips). Images were captured while participants held their breath, in accordance with the American Society of Echocardiography guidelines [25]. LA volumes (LAV) were determined using the biplane area-length technique from both apical four- and two-chamber views and subsequently indexed to body surface area. LAV measurements were taken at the end-systole, immediately before the mitral valve opens (LA maximum volume, LAV_max_); at the end-diastole, right before the mitral valve closes (LA minimum volume, LAV_min_); and at the beginning of the P-wave on the ECG (LAV pre-Atrial contraction, LAV_preA_) [26, 27].

Several parameters were derived from these measurements: LA total emptying volume (LATEV), calculated as LAV_max_ – LAV_min_; LA passive emptying volume (LAPEV), calculated as LAV_max_ – LAV_preA_; LA active emptying volume (LAAEV), calculated as LAV_preA_ – LAV_min_. Corresponding emptying fractions were computed as (LAV/LAV_max_) × 100 for total and passive fractions, and (LAAEV/LAVpreA) × 100 for active emptying fraction.

To assess LV diastolic function, mitral inflow was evaluated using pulsed Doppler with the sample volume positioned between the tips of the mitral leaflets during diastole. Measurements included peak velocities during early diastole (E wave) and late diastole (A wave), the E/A ratio, and deceleration time (DT). Tissue Doppler imaging was used to obtain septal and lateral e' velocities, which allowed calculation of the E/e' ratio as an indicator of LV filling pressure. Isovolumic relaxation time (IVRT) was measured in the apical five-chamber view by placing the pulsed-wave Doppler sample volume in the LV outflow tract to simultaneously visualize the end of aortic ejection and the onset of mitral inflow.

**Fig. 1:**
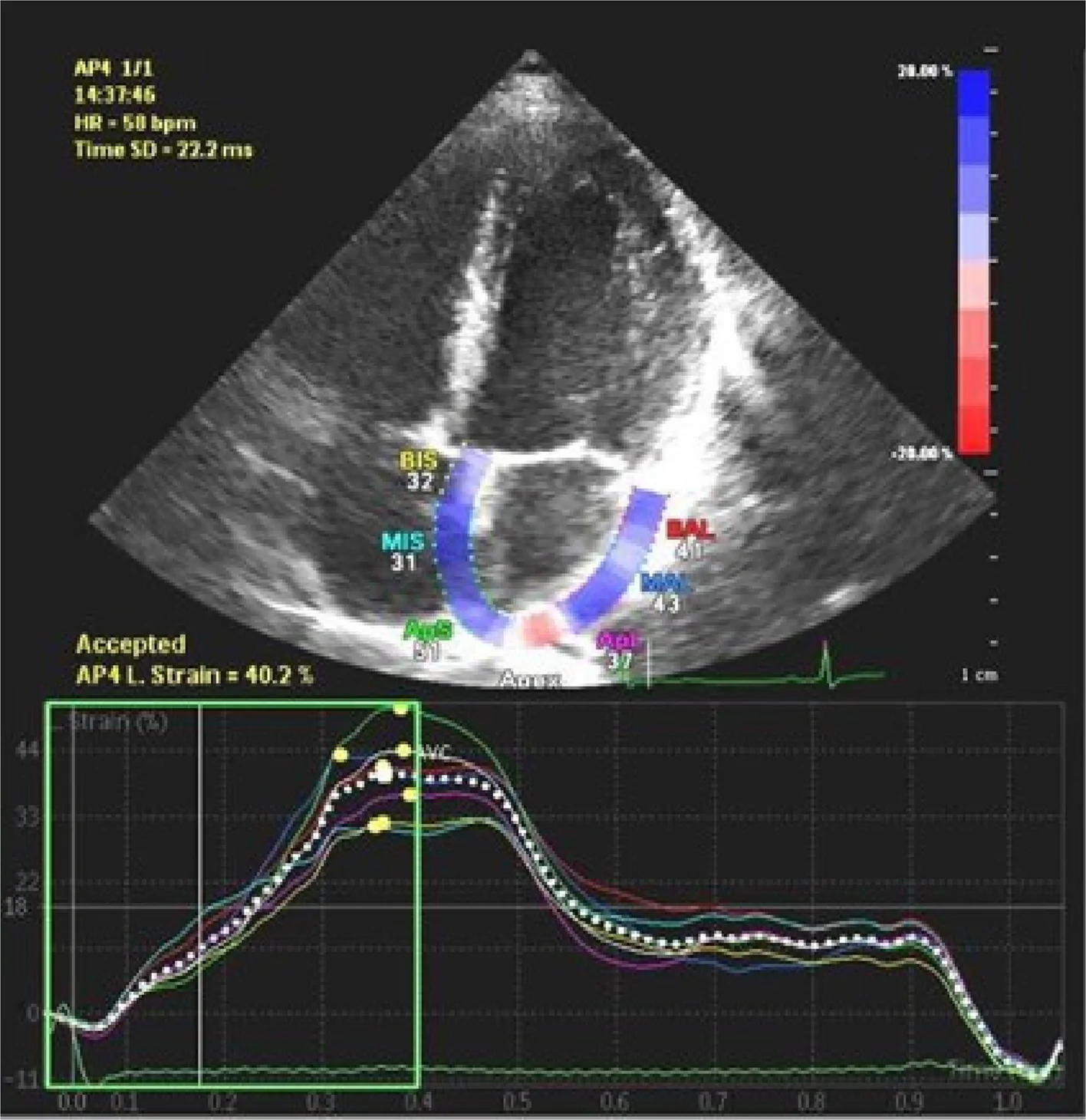
Speckle-tracking echocardiography of the left atrium. AP4, apical four-chamber; ApL, apical anterolateral; ApS, apical inferoseptal; BAL, basal anterolateral; BIS, basal inferoseptal; bpm, beats per minute; HR, heart rate; MAL, mid anterolateral; MIS, mid inferoseptal; ms, milliseconds; SD, standard deviation

STE was also performed for LA imaging. LA strain analysis was performed offline utilizing QLAB ver. 10 (Philips Medical Systems). Two-dimensional grayscale images with a high frame rate (60–80 frames per second) were acquired from apical four- and two-chamber views while participants held their breath. The LA endocardial border was manually traced at ventricular end-systole, excluding pulmonary veins and LA appendage. The region of interest width was adjusted to ensure optimal tracking of atrial myocardium. Automatic frame-by-frame speckle tracking was performed by the software. Manual correction was applied as needed if inadequate tracking occurred (adjusting > 2 segments). Global longitudinal strain of the LA was measured from apical four- and two-chamber views by STE. LA longitudinal strain was assessed in three phases [28]: LA reservoir strain (LASr), the difference between strain at mitral valve opening and ventricular end-diastole; LA conduit strain (LAScd), the difference between strain at the onset of atrial contraction and mitral valve opening; and LA contraction strain (LASct), the difference between strain at ventricular end-diastole and the onset of atrial contraction). Figure 1 shows an example of STE of the LA.

### Statistical analysis

The sample size was determined using G*Power ver. 3.1.9.7 (Heinrich Heine University Düsseldorf). We required 150 patients to detect an effect size of 0.2 for mean STE parameters [29]. The significance level was set at 0.05 with 80% power. Data management and analysis were conducted with IBM SPSS ver. 24.0 (IBM Corp). Descriptive statistics, such as means, standard deviations, medians, ranges, frequencies, and percentages, were reported. The chi-square test was applied to evaluate categorical variables. The normality of continuous variables was checked using either the Kolmogorov–Smirnov or Shapiro–Wilk tests. Differences in means between dichotomous groups were assessed using the Student t-test. One-way analysis of variance compared means across more than two groups, with the Tukey post hoc test for pairwise comparisons. Pearson correlation analysis assessed associations between variables. Statistical significance was set at P < 0.05. One-way analysis of covariance for diastolic mitral indices (E-wave velocity, A-wave velocity, E/A ratio, etc.) comparing between low-, moderate-, and high-risk groups, classified according to CHA_2_DS_2_-VASc score, taking age as a covariate to determine whether differences between risk groups persist after controlling for age.

Multivariable linear regression was used to identify independent predictors of LASr and LAScd among the individual score components. Significant predictors were subsequently included in a hierarchical regression model, with the total CHA_2_DS_2_-VASc score added in the final step. Mediation analysis using the PROCESS macro for SPSS with 5,000 bootstrap samples quantified the proportion of the score's total association with LA strain that was statistically mediated by LV diastolic dysfunction (using average E/e'). The indirect association was deemed significant if the 95% confidence interval excluded zero.

## Results

### Demographics

Participants were categorized into three groups according to their CHA_2_DS_2_-VASc score: low (1 for women, 0 for men), moderate (2 for women, 1 for men), and high (≥ 3 for women, ≥ 2 for men). The ages of the patients ranged from 28 to 82 years, with an average of 45.2 ± 12.3 years. The high-risk group was significantly older than the moderate- and low-risk groups (58.4 ± 10.2 years vs. 42.6 ± 9.4 years vs. 35.2 ± 5.8 years, *P* < 0.001). The cohort had equal sex distribution (75 men, 75 women; 1:1 ratio). The mean body surface area was 1.9 ± 0.1 m^2^. Among participants, one-third had a history of stroke, approximately 40% had hypertension, about 20% had diabetes mellitus, and 4% had vascular disease.

### Correlations between CHA_2_DS_2_-VASc score and echocardiographic parameters

Table 1 summarizes the univariate correlations between total CHA_2_DS_2_-VASc score and various LV and LA parameters. The strongest positive correlations were observed with time-dependent diastolic indices: IVRT (r = 0.714, *P* < 0.001) and DT (r = 0.639, *P* < 0.001). Moderate negative correlations were observed with LAPEV and LATEV (r = –0.533 and r = –0.471, respectively; both *P* < 0.001). There were mild negative correlations with LVEF (r = –0.138, *P* = 0.046), LA passive emptying fraction (LAPEF; r = –0.266, P < 0.001), LAAEV (r = –0.198, *P* = 0.008), and LA total emptying fraction (LATEF; r = –0.171, *P* = 0.018). In contrast, significant, mild positive correlations were seen with LA diameter (r = 0.203, *P* = 0.006), LAV_max_ (r = 0.163, *P* = 0.023), LAV_min_ (r = 0.216, *P* = 0.004), LAV_preA_ (r = 0.255, *P* = 0.003), LAV_min_ index (r = 0.212, *P* = 0.005), and LAV_preA_ index (r = 0.249, *P* = 0.001). There was no significant correlation between the score and LAV_max_ index.

**Table 1:** Correlation between total CHA_2_DS_2_-VASc score and different LV and LA parameters

Parameter	**r**	**P-value**
LVEF (%)	–0.138	**0.046**
LA diameter (cm)	0.203	**0.006**
LAV_max_ (mL)	0.163	**0.023**
LAV_min_ (mL)	0.216	**0.004**
LAV_preA_ (mL)	0.255	**0.003**
LAV_max_ index (mL/m^2^)	0.129	**0.058**
LAV_min_ index (mL/m^**2**^)	0.212	**0.005**
LAV_preA_ index (mL/m^2^)	0.249	**0.001**
**LAPEV (**mL**)**	–0.533	** < 0.001**
**LAPEF** (**%**)	–0.266	** < 0.001**
**LAAEV (**mL**)**	–0.198	**0.008**
**LAAEF** (**%**)	–0.103	**0.105**
**LATEV (**mL**)**	–0.471	** < 0.001**
**LATEF** (**%**)	–0.171	**0.018**
**IVRT (msec)**	0.714	** < 0.001**
**DT (cm/sec)**	0.639	** < 0.001**
STE parameter		
LASr	–0.370	** < 0.001**
LASct	0.045	**0.291**
**LAScd**	–0.428	** < 0.001**
LAScd/LASct	**–0.309**	** < 0.001**

DT, deceleration time; IVRT, isovolumic relaxation time; LA, left atrial; LAAEF, left atrial active emptying fraction; LAAEV, left atrial active emptying volume; LAPEF, left atrial passive emptying function; LAPEV, left atrial passive emptying volume; LAScd, left atrial conduit strain; LASct, left atrial contraction strain; LASr, left atrial reservoir strain; LATEF, left atrial total emptying fraction; LATEV, left atrial total emptying volume; LAV_max_, left atrial maximum volume; LAV_min_, left atrial minimum volume; LAV_preA**,**_ left atrial volume before atrial contraction; LV, left ventricular; LVEF, left ventricular ejection fraction; STE, speckle-tracking echocardiography

Regarding STE parameters, there were significant, moderate negative correlations with LASr (r = –0.370, *P* < 0.001), LAScd (r = –0.428, *P* < 0.001), and LAScd/LASct (r = –0.309, *P* < 0.001).

### Comparison of conventional echocardiographic parameters across CHA_2_DS_2_-VASc risk categories

Table 2 presents comparisons of mitral inflow and tissue Doppler data by risk score. High-risk patients had lower E-wave velocity than those in the low-risk group (68.1 ± 7.6 cm/sec vs. 77.8 ± 11.2 cm/sec, *P* = 0.001). Low-risk patients had lower A-wave velocity (56.8 ± 8.4 cm/sec) compared to moderate-risk (66.5 ± 6.7 cm/sec, P = 0.001) and high-risk patients (67.1 ± 5.9 cm/sec, P = 0.001). Low-risk patients also had a higher E/A ratio (1.4 ± 0.3) than moderate-risk (1.1 ± 0.2, P < 0.001) and high-risk patients (1.1 ± 0.3, P < 0.001). High-risk patients had longer IVRT (96.3 ± 9.1 ms) than moderate-risk (90.7 ± 10.9 ms, P = 0.004) and low-risk patients (87.7 ± 8.6 ms, *P* < 0.001). Low-risk patients had shorter DT (152.9 ± 17.2 ms) compared to moderate-risk (172.2 ± 19.9 ms, P < 0.001) and high-risk patients (165.5 ± 20.5 ms, *P *= 0.007). After age adjustment, differences in E-wave velocity, A-wave velocity, and E/A ratio across CHA_2_DS_2_-VASc groups became nonsignificant (all *P *> 0.05). In contrast, IVRT, DT, average e', and average E/e' remained significantly different after age adjustment (all P < 0.05).

**Table 2:** Mitral inflow and tissue Doppler data comparison in risk score categories (n = 150)

Variable	**CHA**_**2**_**DS**_**2**_**-VASc** risk score category			Pairwise P-value^a^			Overall P-value^**a**^	
	Low (n = 50)	Moderate (n = 50)	High (n = 50)	Low vs. Moderate	Moderate vs. High	Low vs. High	Unadjusted	Age-adjusted
E-wave velocity (cm/sec)	77.8 ± 11.2	72.8 ± 8.8	68.1 ± 7.6	0.095	0.115	0.001^*^	0.006^*^	0.166
A-wave velocity (cm/sec)	56.8 ± 8.4	66.5 ± 6.3	67.1 ± 5.9	0.001^*^	0.840	0.001^*^	0.001^*^	0.122
E/A ratio	1.4 ± 0.3	1.1 ± 0.2	1.1 ± 0.3	< 0.001^*^	0.309	< 0.001^*^	< 0.001^*^	0.083
IVRT (msec)	87.7 ± 8.6	90.7 ± 10.9	96.3 ± 9.1	0.121	0.004^*^	< 0.001^*^	< 0.001^*^	0.018^*^
DT (msec)	152.9 ± 17.2	172.2 ± 19.9	165.5 ± 20.5	< 0.001^*^	0.152	0.007^*^	< 0.001^*^	0.041^*^
Lateral e’ (cm/sec)	16.1 ± 2.6	13.6 ± 3.5	12.0 ± 2.8	< 0.001^*^	0.011^*^	< 0.001^*^	< 0.001^*^	0.011^*^
Average e’	14.3 ± 2.2	11.6 ± 2.4	10.7 ± 1.6	0.001^*^	0.057	< 0.001^*^	< 0.001^*^	0.002^*^
Lateral a’ (cm/sec)	10.1 ± 2.4	11.1 ± 2.7	11.8 ± 2.5	0.048^*^	0.189	0.001^*^	0.005^*^	0.104
Average a’	9.4 ± 1.6	10.2 ± 2.1	10.8 ± 2.4	0.082	0.129	0.001^*^	0.006^*^	0.087
Lateral s’ (cm/sec)	10.5 ± 1.8	9.7 ± 2.1	8.6 ± 1.7	0.051	0.004^*^	< 0.001^*^	< 0.001^*^	0.131
Average s’ (cm/sec)	9.7 ± 1.3	8.9 ± 2.4	8.7 ± 1.8	0.027^*^	0.575	0.006^*^	0.014^*^	0.158
Lateral E/e’	4.9 ± 1.1	5.6 ± 1.3	5.6 ± 1.6	0.039^*^	0.851	0.025^*^	0.045^*^	0.062
Average E/e’	5.5 ± 1.1	6.4 ± 1.2	6.6 ± 1.4	0.005^*^	0.462	< 0.001^*^	0.001^*^	0.038^*^

Values are presented as mean ± standard deviation

DT, deceleration time; IVRT, isovlumetric relaxation time

^a^Post hoc test with Tukey correction was used for pairwise comparisons. ^b^One-way analysis of variance test was used to compare means between groups

^*^P < 0.05

### Comparison of LAV across CHA_2_DS_2_-VASc risk categories

Table 3 compares LVEF and LAV across CHA_2_DS_2_-VASc risk categories. Patients in the high-risk group had a significantly greater LA diameter (3.69 ± 0.40 cm) than those in the moderate-risk (3.50 ± 0.50 cm, P = 0.039) and low-risk groups (3.43 ± 0.1 cm, P = 0.005). High-risk patients also had significantly higher LAV_min_, LAV_min_ index, LAV_preA_, and LAV_preA_ index (14.9 ± 5.1 mL, 8.18 ± 1.8 ml/m^2^, 25.1 ± 7.7 mL, and 13.75 ± 5.5 mL/m^2^, respectively) compared to low-risk patients (12.5 ± 2.6 mL, *P* = 0.006; 6.87 ± 1.6 mL/m^2^, *P* = 0.010; 20.3 ± 5.4 mL, *P* = 0.004; and 11.2 ± 3.1 mL/m^2^, P = 0.006, respectively).

**Table 3:** Ejection fraction, LA dimensions, volumes and strain according to CHA_2_DS_2_-VASc risk score categories

Variable	CHA_2_DS_2_-VASc risk score category			Pairwise P-value^a^			Overall P-value^**a**^
	Low (n = 50)	Moderate (n = 50)	High (n = 50)	Low vs. Moderate	Moderate vs. High	Low vs. High	
LVEF (%)	62.2 ± 3.1	61.8 ± 4.9	60.5 ± 5.5	-	-	-	0.162
LA diameter (cm)	3.4 ± 0.4	3.5 ± 0.5	3.6 ± 0.5	0.436	0.039^*^	0.005^*^	0.016^*^
LAV_max_ (mL)	35.6 ± 8.1	37.6 ± 9.2	39.5 ± 8.5	0.360	0.418	0.086	0.227
LAV_min_ (mL)	12.5 ± 2.6	13.4 ± 4.9	14.9 ± 5.1	0.259	0.088	0.006^*^	0.022^*^
LAV_preA_ (mL)	20.3 ± 5.4	22.7 ± 8.1	25.1 ± 7.7	0.146	0.148	0.004^*^	0.016^*^
LAV_max_ index (mL/m^2^)	19.7 ± 5.6	20.7 ± 6.4	21.0 ± 5.4	0.440	0.823	0.290	0.536
LAV_min_ index (mL/m^2^)	6.9 ± 1.6	7.4 ± 2.6	8.2 ± 1.8	0.320	0.110	0.010^*^	0.034^*^
LAV_preA_ index (mL/m^2^)	11.2 ± 3.1	12.5 ± 4.4	13.7 ± 5.5	0.164	0.168	0.006^*^	0.023^*^
LASr	40.6 ± 5.6	36.9 ± 6.1	33.2 ± 7.1	0.007^*^	0.005^*^	< 0.001^*^	< 0.001^*^
LAScd	26.9 ± 3.9	23.5 ± 4.8	20.3 ± 5.7	0.001^*^	0.003^*^	< 0.001^*^	< 0.001^*^
LASct	13.2 ± 3.3	13.5 ± 4.1	12.9 ± 3.2	0.795	0.413	0.281	0.531
LAScd/LASct	**2.**1** ± 0.6**	**1.9 ± 0.8**	**1.7 ± 0.7**	0.007^*^	0.053	0.023^*^	0.020^*^

Values are presented as mean ± standard deviation

LA, left atrial; LAScd, left atrial conduit strain; LASct, left atrial contraction strain; LASr, left atrial reservoir strain; LAV_max_, left atrial maximum volume; LAV_min_, left atrial minimum volume; LAV_preA**,**_ left atrial volume before atrial contraction; LVEF, left ventricular ejection fraction

^a^Post hoc test with Tukey correction was used for pairwise comparisons. ^b^One-way analysis of variance test was used to compare means between groups

^*^P < 0.05

**Fig. 2:**
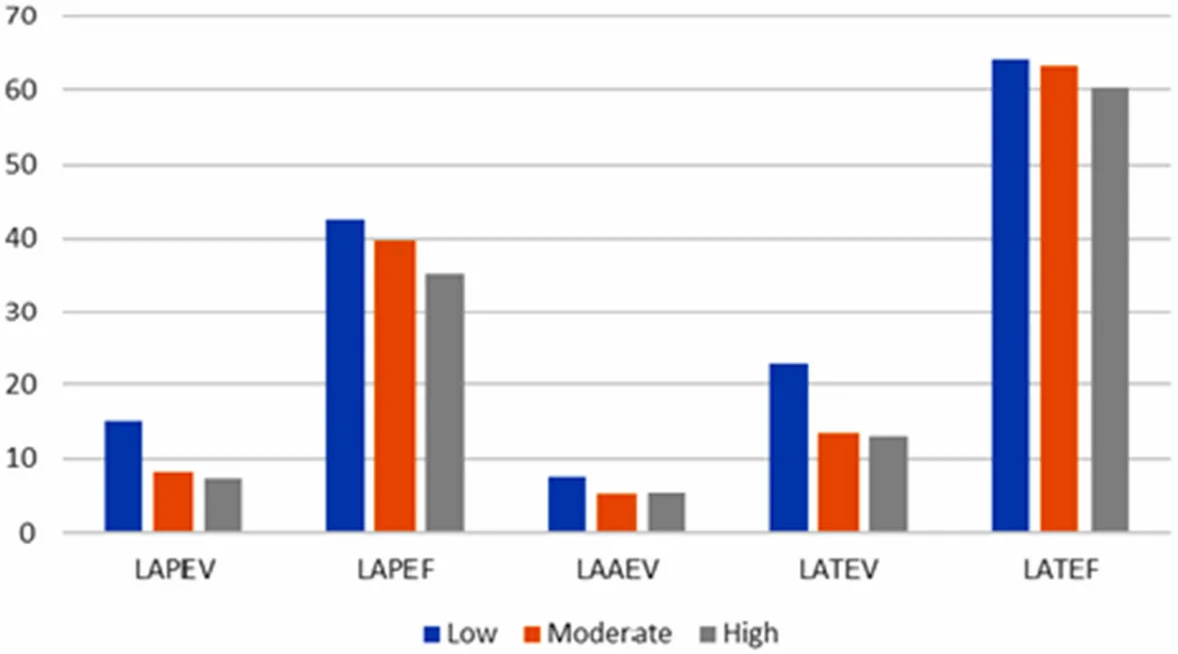
Mean left atrial phasic volumes and functions data comparison in CHA_2_DS_2_-VASc risk score categories. LAAEV, left atrial active emptying volume; LAPEF, left atrial passive emptying fraction; LAPEV, left atrial passive emptying volume; LATEF, left atrial total emptying fraction; LATEV, left atrial total emptying volume

Figure 2 compares phasic LAV by risk group. Low-risk patients had significantly higher LAPEV (15.3 ± 4.1 mL) than those in the moderate-risk (8.3 ± 2.7 mL, P < 0.001) and high-risk groups (7.3 ± 2.8 mL, *P* < 0.001). High-risk patients had significantly lower LAPEF (35.1% ± 9.2%) compared to those in the moderate-risk (39.8% ± 9.1%, P < 0.001) and low-risk groups (42.6% ± 7.8%, *P* < 0.001).

### Comparison of LA strain parameters across CHA_2_DS_2_-VASc risk categories

**Fig. 3:**
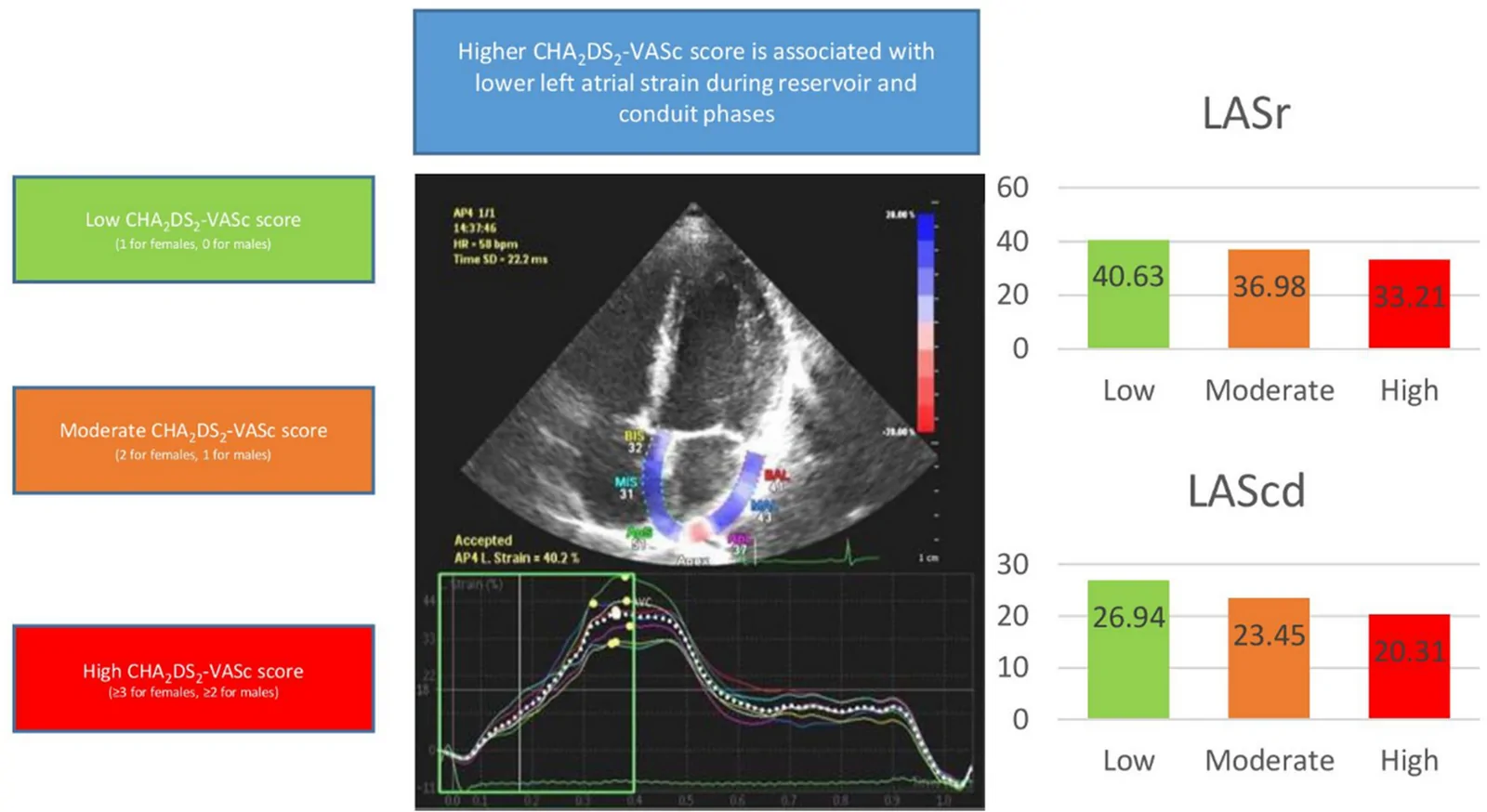
Speckle-tracking echocardiography across different CHA_2_DS_2_-VASc risk score categories shows lower left atrial strain during reservoir and conduit phases with increasing risk score. LAScd, left atrial conduit strain; LASr, left atrial reservoir strain

Table 3 presents a comparison of STE data across risk categories. High-risk patients had markedly lower LASr and LAScd values (33.2 ± 7.1 and 20.3 ± 5.7, respectively) than those in the moderate-risk (LASr: 36.9 ± 6.1, *P* = 0.005; LAScd: 23.5 ± 4.8, *P* = 0.003) and low-risk groups (LASr, 40.6 ± 5.6; LAScd, 26.9 ± 3.9; both *P* < 0.001). The moderate-risk group also had significantly lower LASr and LAScd than the low-risk group (*P* = 0.007 and *P* = 0.001, respectively) (Fig. 3). For LAScd/LASct, low-risk patients had higher values (2.1 ± 0.6) compared to those in the moderate-risk (1.9 ± 0.8, *P* = 0.007) and high-risk groups (1.7 ± 0.7, P = 0.023).

### Regression and mediation analysis

Table 4 presents the independent predictors of LASr and LAScd identified through multivariable regression. Table 5 demonstrates that the total CHA_2_DS_2_-VASc score remained significant after adjustment for its five significant individual components (age, sex, hypertension, heart failure, prior stroke), indicating additive predictive value resulting from combined effects of risk factors. Mediation analysis showed that the association of the score with LAScd was mostly indirect as shown by significant statistical mediation by E/e' (89.7% of total association), with no significant residual direct association (Table 6). For LASr, 62.4% of the association was statistically mediated by E/e', with a significant residual direct score and LASr association remaining (37.6%, β = –0.139, *P* = 0.008).

**Table 4:** Multivariable linear regression analysis for predictors of left atrial strain

Variable	LASr		LAScd	
	β (Standardized)	P-value	β (Standardized)	P-value
Age (per yr)	–0.281	0.002	–0.312	< 0.001
Hypertension (yes vs. no)	–0.168	0.047	–0.184	0.033
Diabetes mellitus (yes vs. no)	–0.145	0.089	–0.198	0.021
Heart failure (yes vs. no)	–0.212	0.011	–0.178	0.038
Prior stroke/TIA (yes vs. no)	–0.226	0.007	–0.245	0.004
Vascular disease (yes vs. no)	–0.098	0.241	–0.087	0.291
Sex (female vs. male)	–0.189	0.024	–0.164	0.049

LAScd, left atrial conduit strain; LASr, left atrial reservoir strain; TIA, transient ischemic attack

**Table 5:** Hierarchical regression analysis: incremental value of CHA_2_DS_2_-VASc total score beyond significant individual components

Step	Variable	Adjusted R^2^		ΔR^2^		*P*-value	
		LASr	LAScd	LASr	LAScd	LASr	LAScd
1	Age	0.118	0.142	-	-	-	-
2	Age + sex	0.142	0.161	+ 0.024	+ 0.019	0.028	0.042
3	Age + sex + hypertension	0.167	0.183	+ 0.025	+ 0.022	0.022	0.031
4	Age + sex + hypertension + heart failure	0.196	0.204	+ 0.029	+ 0.021	0.014	0.035
5	Age + sex + hypertension + heart failure + prior stroke	0.224	0.231	+ 0.028	+ 0.027	0.016	0.018
6	All above + total CHA_2_DS_2_-VASc score	0.249	0.252	+ 0.025	+ 0.021	0.019	0.034

The total CHA_2_DS_2_-VASc score remained significant after adjusting for all five significant individual components (age, sex, hypertension, heart failure, prior stroke), indicating that the aggregated score captures additional predictive information—likely from diabetes and vascular disease (which were nonsignificant individually) as well as additive association of multiple risk factors. This supports the clinical utility of the composite score as an integrated risk marker

LAScd, left atrial conduit strain; LASr, left atrial reservoir strain

**Table 6:** Mediation analysis: decomposition of CHA_2_DS_2_-VASc score association with left atrial strain (mediator: average E/e’)

Pathway	Effect (β)	95% CI^a^	*P*-value	Proportion (%)
LASr				
Total (c)	–0.370	–0.48 to –0.26	< 0.001	100
Indirect (a × b) via E/e’	–0.231	–0.32 to –0.14	< 0.001	62.4
Direct (c')	–0.139	–0.24 to –0.04	0.008	37.6
LAScd				
Total (c)	–0.428	–0.54 to –0.32	< 0.001	100
Indirect (a × b) via E/e’	–0.384	–0.48 to –0.29	< 0.001	89.7
Direct (c')	–0.044	–0.14 to + 0.05	0.342	10.3

CI, confidence interval; LAScd, left atrial conduit strain; LASr, left atrial reservoir strain; TIA, transient ischemic attack

^a^Bootstrapping with 5,000 resamples was used for 95% CIs

## Discussion

This study found that elevated CHA_2_DS_2_-VASc scores were linked to gradual worsening of LV diastolic function, as well as reduced LA reservoir and conduit function, while contractile function (measured by STE) remained largely unchanged. Mediation analysis suggests that the score's statistical association with LA strain is primarily indirect, operating through LV diastolic dysfunction.

The CHA_2_DS_2_-VASc score was originally designed to assess the risk of thromboembolism in individuals with AF. More recently, its usefulness has been explored in populations without AF, such as patients with heart failure, chronic obstructive pulmonary disease, and those who have undergone CABG [7–10]. Despite this, it remains unclear whether the score can reliably detect LA dysfunction in people with sinus rhythm. The current study aimed to investigate the CHA_2_DS_2_-VASc score as a potential early marker for LA dysfunction in this group.

In this study, a mild but statistically significant inverse relationship was observed between the CHA_2_DS_2_-VASc score and LVEF, although no significant differences in LVEF were detected across the three groups. Notably, patients with LVEF below 50% were excluded from the analysis. Previous research has demonstrated that higher CHA_2_DS_2_-VASc scores are associated with declining LVEF in individuals with CAD [30, 31]. Regarding mitral annular systolic velocity (s'), univariate analysis demonstrated significantly lower s’ velocities in the high- and moderate-risk groups compared to the low-risk group, consistent with the findings of Hadadi et al. [30], who reported a marked reduction in mean mitral s’ wave velocity in high-risk patients. However, after adjusting for age, the differences in s' wave velocities were no longer statistically significant. This suggests that the observed decline in s' velocities across CHA_2_DS_2_-VASc risk groups is primarily explained by physiological aging rather than disease-specific systolic dysfunction. This interpretation is further supported by the exclusion of patients with LVEF < 50%, which eliminated overt systolic impairment from the analysis and identified age as the dominant determinant of s' variation. Therefore, while mitral s' velocity may serve as a marker of subtle LV systolic dysfunction in selected populations [32], its utility in the context of CHA_2_DS_2_-VASc risk stratification is confounded by age and should be interpreted with caution.

The present study demonstrated that LV diastolic function was impaired in both high- and moderate-risk groups compared to the low-risk group. Conventional pulsed-wave Doppler analysis identified a strong positive correlation between DT (r = 0.639) and IVRT (r = 0.714) and the overall CHA_2_DS_2_-VASc score, as well as a moderate, significant negative correlation with the E/A ratio. Following adjustment for age, mitral inflow parameters (E-wave velocity, A-wave velocity, E/A ratio) no longer differed significantly among risk groups. These findings suggest that univariate differences in these parameters are attributable to physiological aging rather than diastolic dysfunction. In contrast, IVRT, DT, e', and E/e' remained significant after age adjustment, supporting their utility as robust markers of pathological diastolic dysfunction. Hadadi et al. [30] reported a significant decrease in the E/A ratio with increasing CHA_2_DS_2_-VASc scores, although significant DT prolongation was not observed. Similarly, Azarbal et al. [31] described this trend in patients with stable CAD and preserved ejection fraction. Discrepancies with the findings of Hadadi et al. [30] regarding the E/A ratio likely result from their lack of age adjustment. In the present study, adjustment for age rendered the E/A ratio nonsignificant, while other diastolic indices remained significant.

Regarding tissue Doppler indices, a mild, significant positive correlation was observed between the average E/e' and the total CHA_2_DS_2_-VASc score, while a moderate, significant negative correlation was found with the average e'. These findings are consistent with those of Hadadi et al. [30], who reported a decrease in e' and an elevation of E/e' with increasing CHA_2_DS_2_-VASc score. Jang et al. [33] also demonstrated that increasing CHA_2_DS_2_-VASc score was associated with higher LAV index and E/e' in patients with nonvalvular AF.

Speckle-tracking analysis of the LA showed a moderate, significant inverse relationship between LASr, LAScd, LAScd/LASct, and the CHA_2_DS_2_-VASc score. These measures were notably lower in the high-risk group compared to both the moderate- and low-risk groups. This observation is consistent with the findings of Hadadi et al. [30], who documented decreased LA strain and strain rate during the reservoir phase in individuals with higher risk scores. The various components of the CHA_2_DS_2_-VASc score may impact LA function through different mechanisms, including myocardial fibrosis, insulin resistance, oxidative stress related to diabetes, age-associated fibrosis, hypertension, estrogen deficiency following menopause, neurohormonal activation in heart failure, and other processes seen in patients with myocardial infarction and stroke [34–37]. The total CHA_2_DS_2_-VASc score showed additive predictive value after adjusting for its significant individual components. These findings may support the clinical use of the CHA_2_DS_2_-VASc score as an integrated risk assessment tool, rather than depending on its individual components.

Unlike previous studies that only demonstrated association, the present mediation analysis estimated the association of the CHA_2_DS_2_-VASc score with LA strain. For LAScd, 89.7% of the total association with the score was statistically mediated by LV diastolic dysfunction. This finding suggests that low conduit strain in high-risk patients primarily reflects elevated LV filling pressure rather than primary atrial disease. For LASr, 62.4% of the association was statistically mediated by LV diastolic dysfunction, with a residual significant direct association remaining (β = –0.139, P = 0.008). This result suggests that a possible component of intrinsic atrial pathology, such as fibrosis or altered compliance, not explained by ventricular dysfunction.

Volumetric assessment supported the LA strain findings. Mild, significant positive correlations were observed between LA diameter, LAV_max_, LAV_min_, LAV_min_ index, LAV_preA_, and the CHA_2_DS_2_-VASc score. There was no significant correlation between CHA_2_DS_2_-VASc score and LAV_max_ index. Hadadi et al. [30] similarly reported increases in LAV_max_ index, LAV_preA_, and LAV_min_ index across score groups. The high-risk group demonstrated worsening of reservoir function (lower LATEV and LATEF) and conduit function (lower LAPEV and LAPEF) compared to the low-risk group, with no significant difference in contractile function (LAAEV and LA active emptying fraction [LAAEF]). Hadadi et al. [30] also found reduced LATEF, while reductions in LAPEF and LAAEF were not significant.

Although this study is cross-sectional, a growing body of evidence has established the prognostic value of LA strain. A meta-analysis of 5,767 heart failure patients showed that each 1% decrease in LA reservoir strain was associated with a 5% increase in mortality or hospitalization. Another meta-analysis of eight studies (17,223 patients) found each 1% reduction in LASr was associated with a 13% increase in incident stroke. Following transcatheter aortic valve replacement, LASr below 15.5% was associated with a 1.7-fold increase in 3-year mortality. These published associations support the potential clinical relevance of these findings [38–40].

### Limitations

The cross-sectional design prevents causal inference; longitudinal studies are needed to determine whether reduced LA strain predicts new-onset AF or thromboembolism. LA strain was measured using LV-dedicated software (due to availability), which may affect reproducibility, though all measurements used the same platform consistently. The study's findings could be enhanced by incorporating three-dimensional echocardiography, cardiac magnetic resonance imaging, invasive LV filling pressure measurements, or Holter monitoring. The study was designed before the introduction of the CHA_2_DS_2_-VA score (which removed the sex category), which was updated by recent guidelines.

## Conclusions

Patients with higher CHA_2_DS_2_-VASc risk scores and sinus rhythm generally demonstrate lower LA strain as measured by STE. These patients also exhibit impaired LV diastolic function and increased LA phasic volumes, with reduced phasic emptying except for a compensatory increase in contractile function. Reduced LA strain is largely accounted for by LV diastolic dysfunction, especially during the conduit phase. The CHA_2_DS_2_-VASc retains residual direct association with the reservoir phase of LA strain, suggesting possible atrial-specific pathology. Interventions targeting LV diastolic dysfunction—blood pressure control, glycemic management, heart failure treatment—may secondarily improve LA function.

## Data Availability

The data that support the findings of this study are available from the authors upon request.

## Abbreviations

AF: Atrial fibrillation
CABG: Coronary artery bypass graft
CAD: Coronary artery disease
DT: Deceleration time
ECG: Electrocardiogram
IVRT: Isovolumic relaxation time
LA: Left atrium
LAAEF: Left atrial active emptying function
LAAEV: Left atrial active emptying volume
LAPEF: Left atrial passive emptying function
LAPEV: Left atrial passive emptying volume
LAScd: Left atrial conduit strain
LASct: Left atrial contraction strain
LASr: Left atrial reservoir strain
LATEF: Left atrial total emptying fraction
LATEV: Left atrial total emptying volume
LAV: Left atrial volumes
LAV_max_: Left atrial maximum volume
LAV_minx_: Left atrial minimum volume
LAV_preA_: Left atrial volume before atrial contraction
LV: Left ventricular
LVEF: Left ventricular ejection fraction
STE: Speckle-tracking echocardiography
STROBE: Strengthening the Reporting of Observational Studies in Epidemiology
